# The frequency of adherence, *biofilm-associated*, Arginine Catabolic Mobile element genes, and biofilm formation in clinical and healthcare worker coagulase-negative staphylococci isolates

**DOI:** 10.1186/s12866-023-02959-x

**Published:** 2023-08-15

**Authors:** Davood Kalantar-Neyestanaki, Shahla Mansouri, Omid Tadjrobehkar, Elham Isaei

**Affiliations:** 1 Medical Mycology and Bacteriology Research Center, Kerman University of Medical Sciences, Kerman, Iran; 2 Department of Medical Microbiology (Bacteriology and Virology), Afzalipour Faculty of Medicine, Kerman University of Medical Sciences, Kerman, Iran; 3 Noncommunicable Diseases Research Center, Bam University Of Medical Sciences, Bam, Iran

**Keywords:** Biofilm, Coagulase-negative *Staphylococci*, Healthcare workers, Virulence and biofilm-associated genes

## Abstract

**Background:**

Healthcare workers may pave the way for increased infections in hospitalized patients by coagulase-negative staphylococci (CoNS). Biofilm formation and antibiotic resistance are the major problems posed by CoNS in nosocomial infections. In this study, we determined biofilm production level and the distribution of biofilm-associated and virulence genes, including *icaADBC, aap, bhp, atlE, embp*, and *fbe*, as well as IS*256*, IS*257*, *mecA*, and ACME clusters (*arc-A, opp-3*AB) among 114 clinical (n = 57) and healthcare workers (n = 57) CoNS isolates in Kerman, Iran.

**Results:**

In this study, more than 80% (n = 96) of isolates were methicillin-resistant CoNS (MR-CoNS). Out of 114 isolates, 33% (n = 38) were strong biofilm producers. Strong biofilm formation was found to be significantly different between clinical and healthcare workers’ isolates (*P* < 0.050). In addition, 28% (n = 32) of isolates were positive for *icaADBC* simultaneously, and all were strong biofilm producers. The prevalence of *icaADBC, mecA, bhp, fbe*, and IS*256* in clinical isolates was higher than that in healthcare workers’ isolates (*P* < 0.050). A significant relationship was observed between clinical isolates and the presence of *icaADBC, mecA*, *bhp*, and IS*256*. Although these elements were detected in healthcare workers’ isolates, they were more frequent in clinical isolates compared to those of healthcare workers.

**Conclusions:**

The high prevalence of ACME clusters in healthcare workers’ isolates and biofilm formation of these isolates partially confirms the bacterial colonization in the skin of healthcare workers. Isolating MR-CoNS from healthcare workers’ skin through similar genetic elements to clinical isolates, such as *icaADBC*, *mecA*, and IS*256*, calls for appropriate strategies to control and prevent hospital infections.

**Supplementary Information:**

The online version contains supplementary material available at 10.1186/s12866-023-02959-x.

## Introduction

Given the increased infection risk factors in hospitalized patients, it is crucial to understand the presence of virulence genes and the prevalence of antibiotic resistance among bacteria that cause nosocomial infections [1]. Coagulase-negative staphylococci (CoNS) isolates are important opportunistic pathogens among hospitalized patients. In addition, Staphylococcus isolates are one of the major skin and mucous membrane microbiota [2, 3]. According to a previous study, CoNS isolates can spread among healthcare workers and hospitalized patients [3–6].

Various genes, including *icaADBC* (intracellular adhesion) operon, *fbe* (fibrinogen binding protein), *bhp* (bap homologous- protein), *embp* (extracellular matrix-binding protein), *aap* (accumulation-associated protein), and *atlE* (autolysin), are involved in biofilm formation, mediate the initial adhesion, and bind specifically to fibrinogen, fibronectin, and collagen. The *ica* operon can be regulated by insertion sequence (IS) elements. The integration of IS elements can alter genes expression, and IS*256* confers phase variation phenomena concerning biofilm formation, modulation of antibiotic resistance and virulence genes in *S. aureus* and CoNS isolates [7–11].

Biofilms has considerably affect on evasion of host immune system and resistance to antibiotics in staphylococcal infections [12–14]. Biofilm formation and colonization on medical devices make the CoNS isolates one of the most frequent causes of nosocomial infections in hospitalized patients [12–14]. Nowadays, most clinical CoNS isolates, such as *S. epidermidis* and *S. haemolyticus*, are methicillin-resistant (MR-CoNS) and are commonly resistant to different antibiotic agents [3, 14].

Typically, the biofilm formation in CoNS isolates is based on the polysaccharide intercellular adhesion (PIA) matrix, which is also known as poly-N-acetyl-glucosamine (PNAG). This polysaccharide matrix is synthesized with proteins encoded by the *icaADBC* operon in *Staphylococci* species [12, 15]. On the other hand, the microbial surface components, which recognize adhesive matrix molecules (MSCRAMMs), such as Fbe and Embp, can bind to fibrinogen and fibronectin, respectively, and have an important role in attachment to the cells and biofilm production in CoNS [13]. Also, other virulence genes, such as *atlE*, *bhp*, and *aap*, are commonly found in *S. epidermidis* isolates and act as initial attachment factors in biofilm formation [6, 7]. Identifying and targeting polysaccharides and proteins involved in adhesion and biofilm formation can be suitable candidates for vaccine production that prevent staphylococcal infections [16–18]. The vaccine can be effective in preventing the infection. Although there is a good body of research on this issue, further practical studies should be undertaken on the vaccine to prevent infections [16–18].

The arginine catabolic mobile element (ACME) consists of two gene clusters: the *arc*-operon encoding a secondary arginine deaminase system and the *opp3*-operon encoding a putative oligopeptide permease system [19, 20]. These elements are homologs of virulence determinants in other bacterial species and enhance bacterial adaptability and colonization [20, 21]. The horizontal transfer of the ACME in intra-species of Staphylococci was confirmed in previous studies [19, 20]. However, the ACME was shown to contribute to the colonization and survival of CoNS isolates in hospital settings. ACME allotypes were classified as (1) ACME-I containing the *arc* and the *opp-3* gene clusters, (2) ACME-II containing the *arc*, and (3) ACME-III containing the *opp-3* [20, 22]. The transmission of infectious agents by healthcare workers was observed in hospital settings [2, 3]. Therefore, it is important to study the characteristics of typical flora isolates from hospital staff to control infection in hospitals. The present study determines biofilm formation ability and the prevalence of biofilm-associated and virulence genes, IS*256*, IS*257*, *mecA*, and ACME clusters in CoNS isolates collected from clinical staff and healthcare workers’ skin in Kerman, Iran

## Results

### Bacterial strains and methicillin-resistant CoNS detection

In total, 114 CoNS isolates were collected in this study. Different species of CoNS isolates, including *S. epidermidis* 77.2% (n = 88), *S. haemolyticus* 16.6% (n = 19), *S. hominis* 5% (n = 6), and *S. saprophiticus* 1*%* (n = 1), were detected. Among 114 clinical and healthcare workers’ CoNS isolates, 66.5% (n = 76), 10.5% (n = 12), 20% (n = 23), 6% (n = 7), 3.6% (n = 4), and 1.8% (n = 2) were collected from neonatal intensive care units (NICU), intensive care unit (ICU), infectious disease units (ID), pediatric intensive care units (PICU), bone marrow transplant (BMT), and oncology units, respectively. In addition, 96.5% (n = 56) of clinical and 70% (n = 40) of healthcare workers’ isolates were resistant to cefoxitin and considered MR-CoNS. All MR-CoNS isolates were positive for *mecA*.

### Biofilm assay

Biofilm formation was observed at strong, moderate, and weak levels in 96.5% (n = 55) of clinical and 91% (n = 52) of healthcare workers’ isolates in this study. Among clinical samples, 46% (n = 26) of isolates were categorized as strong biofilm producers, 37% (n = 21) were moderate biofilm producers, 14% (n = 8) were weak biofilm producers, and two isolates were non-biofilm producers. In healthcare workers’ isolates, 21% (n = 12) were strong biofilm forming, 30% (n = 17) were moderate biofilm forming, 40% (n = 23) were weak biofilm forming, and 9% (n = 5) isolates were non-biofilm forming. Also, the prevalence of strong biofilm formation in clinical isolates was higher than that of healthcare workers’ isolates (*P* < 0.004). Among 88 *S. epidermidis*, 40% (n = 36), 23% (n = 20), and 36% (n = 32) were strong, moderate, and weak biofilm producers, respectively. Moreover, among 19 *S. heamolyticus*, 89% (n = 17) and 10% (n = 2) were moderate and weak biofilm producers, respectively. Two *S. hominis* were strong, and 3 were moderate biofilm producers.

### Detection of ***ica*** operon, ACME type, IS, and virulence genes

**Fig. 1:**
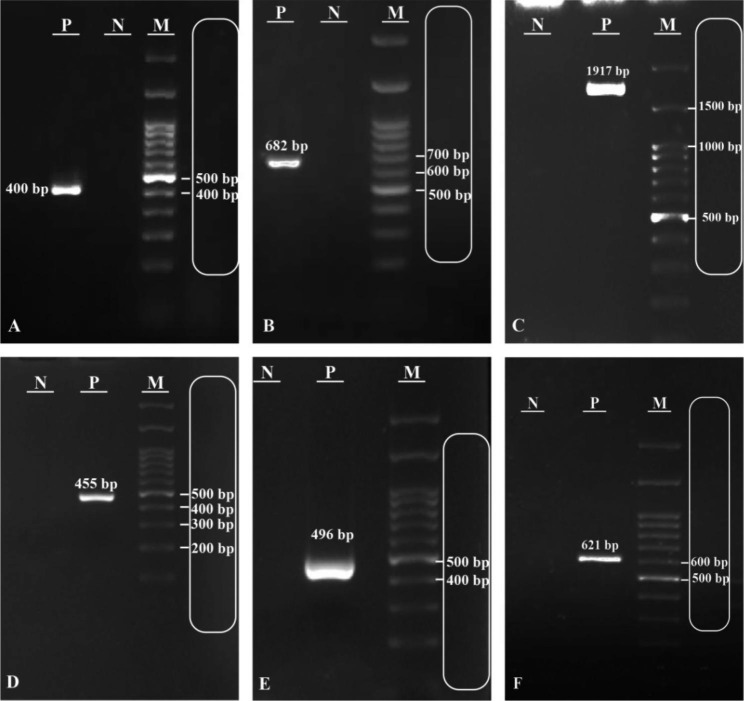
PCR electrophoresis: **A**: *aap* (400 bp), **B**: *atlE* (682 bp), **C**: *bhp* (1917 bp), **D**: *embp* (455 bp), **E**: *fbe* (496 bp) and **F**: IS*257* (621 bp) **F**: M: 100 bp DNA marker, **P**: Positive control, **N**: Negative control

The profile of *icaADBC*, virulence genes, and methicillin resistance among biofilm producer of CoNS isolates are presented in Tables 1 and 2. The rate of *icaA* and *icaB* was 33% (n = 38), while the prevalence of *icaC* and *icaD* was 34% (n = 39) and 37.7% (n = 43), respectively. In clinical isolates, 42% (n = 24) were positive for *icaADBC*, while 17% (n = 10) of healthcare workers’ isolates were positive for this operon. There was a significant relationship between *icaD* and *mecA* in both groups of isolates, among 42 isolates were positive for *icaD*, 93% (n = 39) were positive for *mecA* simultaneously (*P* < 0.030). The *fbe* and *bhp* were detected in 11.4% (n = 17) and 6% (n = 7) of isolates, respectively. In addition, the prevalence of *embp*, *aap*, and *atlE* was found to be 55% (n = 63), 53.5% (n = 61), and 51% (n = 58) in isolates, respectively as shown in Table 3 (Fig. 1).

**Table 1:** The profile of biofilm-associated and virulence genes among 57 clinical isolates

Isolates (n)	Samples (n)	Biofilm (n)	MR or MS isolates (n)	ACME-type (n)	Genes profile
*S. epidermidis* (1)	CSF	S	MR	*-*	*icaADBC, bhp, atlE*
*S. epidermidis*(1)	WI	S	MR	*-*	*icaADBC, bhp, aap*
*S. epidermidis* (1)	BSI	S	MR	*-*	*icaAD, aap*
*S. epidermidis*(1)	BSI	S	MR	*-*	*icaADBC, bhp, aap, embp*
*S. epidermidis*(1)	BSI	S	MR	*-*	*icaADBC,fbe, bhp, aap, embp, atlE*
*S. epidermidis*(1)	BSI	S	MR		*bhp, embp, aap*
*S. epidermidis*(3)	BSI(2), WI(1)	S	MR	II (2)	*icaADBC, embp, aap, atlE*
*S. epidermidis*(2)	BSI(1),CSF(1)	S	MR	-	*icaADBC*
*S. epidermidis*(1)	WI	S	MR	-	*icaADBC, embp*
*S. hominis*(1)	WI	S	MR	-	*icaADBC, embp, aap*
*S. epidermidis*(1)	WI	S	MR	III (1)	*icaADBC, embp, aap*
*S. epidermidis*(2)	BSI(1)	S	MR	-	icaADBC, embp, atlE
*S. epidermidis*(2)	BSI	S	MR	-	*icaADBC, fbe, embp, atlE*
*S. epidermidis*(4)	BSI	S	MR	І (2)	*icaADBC, fbe, embp, atlE,aap*
*S. hominis*(1)	BSI	S	MR	-	*icaADBC, embp*
*S. epidermidis*(1)	BSI	S	MR	І	*icaAD, fbe, embp, atlE*
*S. epidermidis*(1)	BSI	S	MS	-	*icaC, fbe, aap, atlE,embp*
*S. epidermidis*(1)	WI	S	MR	-	*IcaADBC, aap*
*S. hominis*(1)	BSI	M	MR	-	*IcaAD, aap*
*S. epidermidis*(1)	BSI	M	MR	III	*embp, aap, atlE*
*S. epidermidis*(1)	BSI	M	MR	-	*icaDB*
*S. epidermidis*(1)	CSF	M	MR	-	*IcaADB, aap, embp, atlE*
*S. epidermidis*(3)	BSI	M	MR	-	*aap*
*S. heamolyticus*(1)	BSI	M	MR	-	*icaD,aap*
*S. epidermidis*(1)	WI	M	MR	-	*IcaD,embp, aap, atlE*
*S. epidermidis*(1)	BSI	M	MR	-	*embp,aap, atlE*
*S. epidermidis*(1)	BSI	M	MS	-	*embp, aap*
*S. epidermidis*(1)	BSI	M	MR	-	*embp*
*S. epidermidis*(1)	BSI	M	MR	-	*Bhp, fbe, embp, aap*
*S. epidermidis*(3)	BSI	M	MR	III (1)	*fbe, embp, atlE, aap*
*S. epidermidis*(2)	BSI	M	MR	-	*aap, atlE*
*S.heamolyticus*(1)	BSI	M	MR	-	*embp, aap*
*S. saprophiticus*(1)	BSI	M	MR	-	*aap, atlE*
*S. epidermidis*(4)	BSI (3), WI (1)	W (3), M (1)	MR	-	*-*
*S. heamolyticus*(2)	BSI	W	MR	-	*embp, atlE*
*S. epidermidis*(1)	BSI	W	MR	*-*	*IcaD, atlE*
*S. epidermidis*(1)	BSI	W	MR	*-*	*atlE, embp*
*S. heamolyticus*(1)	BSI	W	MR	-	*-*

BSI: Blood stream infection, CSF: Cerebrospinal fluid, WI: Wound infection, S: Strong biofilm producer, M: Moderate biofilm producer, W: Weak biofilm producer, MR: Methicillin resistance, MS: Methicillin sensitive, n: Number of isolates

**Table 2:** The profile of biofilm-associated and virulence genes among 57 healthcare worker isolates isolates

Isolates (n)	Biofilm(n)	MR or MS isolates (n)	ACME-type (n)	Genes profile
*S. epidermidis* (5)	S	MR(4), MS(1)	III (3)	*icaADBC, embp, aap, atlE*
*S. epidermidis*(1)	S	MR	III	*icaADBC, aap*
*S. epidermidis*(2)	S	MR(1), MS(1)	III (1)	*IcaADBC, embp, aap*
*S. epidermidis*(1)	S	MR	II	*icaADBC, aap, atlE*
*S. epidermidis*(1)	S	MS	-	*icaADBC, atlE*
*S. epidermidis*(2)	S	MR	II (1)	*embp, aap*
*S. epidermidis*(5)	M	MR	І (1), III (1)	*embp, atlE*
*S. epidermidis*(5)	M	MR	-	*embp, aap,atlE*
*S. hominis*(1)	M	MR	III	*IcaC, embp, aap*
*S. epidermidis*(2)	M	MR	II (1)	*IcaC, embp, aap*
*S. heamolyticus*(1)	M	MR	III	*aap*
*S. heamolyticus*(1)	M	MR	-	*embp,aap*
*S. epidermidis*(5)	W	MS(3), MR(2)	І (1), III (1)	*-*
*S. epidermidis*(1)	W	MR	-	*icaBC*
*S.heamolyticus*(1)	W	MS	-	*embp*
*S. heamolyticus*(3)	W	MR	II (2), III (1)	*embp, atlE*
*S. heamolyticus*(2)	W	MR	II (1), III (1)	*aap*
*S. heamolyticus*(3)	W	MR	II (1)	*atlE*
*S. epidermidis*(1)	W	MR	-	*icaBC*
*S. hominis*(1)	W	MR	-	*-*
*S. epidermidis(3)*	W	MR	II (1)	*aap*
*S. epidermidis*(5)	M(2),W(3)	MR(4), MS(1)	III (2)	*embp, aap, atlE*

S: Strong biofilm producer, M: Moderate biofilm producer, W: Weak biofilm producer, MR: Methicillin resistance, MS: Methicillin sensitive, n: Number of isolates

We did not observe a significant difference between clinical and healthcare workers’ isolates in terms of *embp*, *aap*, and *atlE* prevalence. The prevalence of IS*257* was higher than IS*256* in both clinical and healthcare workers’ isolates (83% vs. 46%). The frequency of *fbe* (23% vs. 7%) and IS*256* (79% vs. 14%) in clinical isolates was higher than the one in healthcare workers’ isolates (*P* < 0.050). The distribution of *icaA*, *icaB*, *icaC*, *icaD*, and biofilm-associated virulence genes (*fbe, bhp, embp, aap*, and *atlE*) in clinical and healthcare workers’ isolates are presented in Table 3.

**Table 3:** Prevalence of biofilm associated, virulence, *mecA* genes, IS*256*, IS*257* and ACME cluster genes among clinical and healthcare worker´ isolates

Genes	Number (%) of isolates			
	Total of isolates n = 114	Healthcare worker isolates n = 57	Clinical isolates n = 57	*P-value*
***icaA***	38 (33)	12 (21)	26 (45.6)	0.009
***icaB***	38 (33)	14 (24.5)	24 (42)	0.073
***icaC***	39 (34)	16 (28)	23 (40)	0.000
***icaD***	42 (37.7)	12 (21)	30 (52.6)	0.000
***fbe***	13 (11.4)	0	13 (23)	0.000
***bhp***	7 (6)	(0)	7 (12)	0.013
***embp***	63 (55)	30 (52.6)	33 (59)	0.053
***aap***	61 (53.5)	29 (51)	34 (56)	0.088
***atlE***	58 (51)	28 (49)	31(52.6)	0.053
***mecA***	95 (83.3)	40 (70)	55 (96.5)	0.000
***IS256***	53 (46)	8 (14)	45 (79)	0.000
***IS257***	95 (83)	45 (79)	50 (88)	0.091
***arcA***	16 (14)	11 (19)	5 (8)	0.000
***opp3AB***	19 (16)	14 (24.5)	5 (8)	0.000

Among 37 strong biofilm producers, 32 isolates were positive for *icaADBC*, and we observed a significant relationship between the presence of *icaADBC* operon and strong biofilm production in isolates (*P* < 0.001). Furthermore, in moderate and weak biofilm-forming isolates, the frequency of *icaADBC* was lower compared to strong biofilm-producing isolates. The prevalence of *aap* in the moderate biofilm producers (79%, 30 out of 38) was higher compared with other isolates. Biofilm-associated genes among the biofilm-producing clinical and healthcare workers’ isolates are presented in Table 4. *Fbe*, *bhp*, *embp*, and IS*256* were more frequently present in *icaAD-*positive isolates than in *icaAD-*negative isolates (*P* < 0.030).

**Table 4:** Prevalence of the Biofilm-associated genes and IS elements (IS*256* and IS*257*) among the strong (n = 38), moderate (n = 38), and weak (n = 31) biofilm producers. P1: p-value between strong biofilm and moderate biofilm isolates. P2: p-value between strong biofilm and weak biofilm isolates. P3: p-value between moderate biofilm and weak biofilm isolates

Biofilm associated genes	Strong biofilm isolates n (%)	Moderate biofilm isolates n (%)	Weak biofilm isolates n (%)	P1	P2	P3
***icaA***	38 (100)	2 (5)	0	0.000	0.000	0.000
***icaB***	32 (84)	2 (5)	4 (13)	0.000	0.000	0.000
***icaC***	33 (86)	3 (8)	3 (10)	0.000	0.000	1.000
***icaD***	36 (95)	5 (13)	1(3)	0.000	0.000	0.033
***fbe***	9 (24)	4 (10.5)	0	0.000	0.003	0.059
***bhp***	6 (16)	1(3)	0	0.000	0.028	1.000
***aap***	26 (68)	30 (79)	7 (22.5)	0.000	0.001	0.000
***atlE***	23 (60.5)	21 (55)	15 (48)	0.053	0.000	0.000
***embp***	27 (71)	25 (66)	9 (29)	1.000	0.002	0.004
***arcA***	5 (13)	6 (16)	5 (16)	1.000	1.000	1.000
***oppAB***	9 (24)	5 (13)	6 (19)	0.000	0.060	1.000
***IS256***	23 (61)	21 (55)	15(48)	0.001	0.000	0.000
***IS257***	36 (95)	31 (82)	23 (74)	0.056	0.009	0.080

**Fig. 2:**
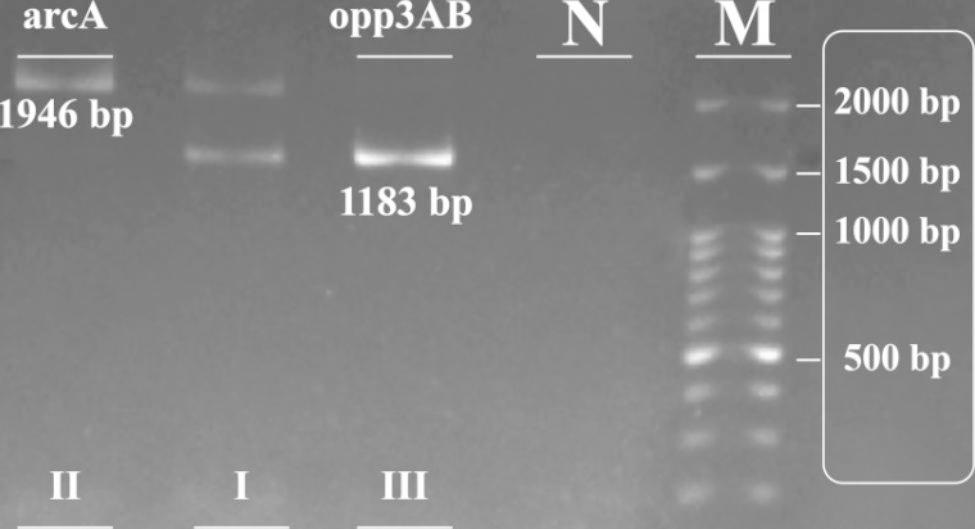
PCR electrophoresis: *opp3AB (1183 bp)* and *arcA* (1946 bp), M: 100 bp DNA marker. І:ACME- І, II: ACME- II and III: ACME- III. N: Negative control

**Fig. 3:**
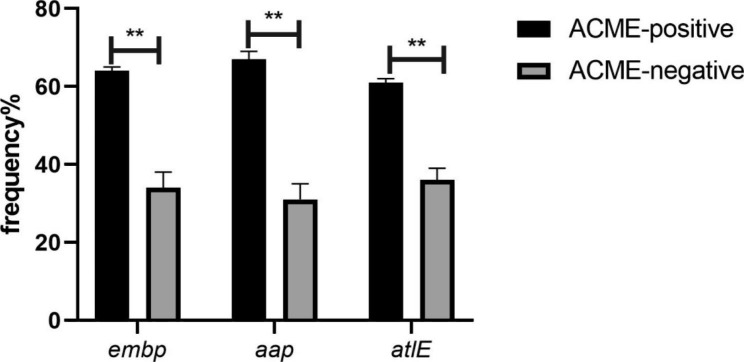
The prevalence of *embp, aap*, and *atlE* in ACME positive and ACME negative isolates. (**: P-Value < 0.010)

In total, 31 isolates were positive for ACME clusters, including 40% (n = 23) of healthcare workers’ isolates and 14% (n = 8) of clinical isolates. Among the 23 healthcare workers’ ACME-positive isolates, 50% (n = 12) harbored ACME-III (*opp-3AB* positive), 39% (n = 9) harbored ACME-II (*arcA* positive), and 8.6% (n = 2) were ACME-І (*arcA* and *opp-3AB* positive). In addition, 8 clinical isolates were positive for ACME clusters, among which 37% (n = 3) were ACME-І and ACME-III, and 25% (n = 2) were ACME-II (Fig. 2). The frequency of ACME in healthcare workers’ isolates was considerably higher than that in clinical isolates (*P* < 0.001) (Table 3). We should note that the isolates with ACME were biofilm producers (strong, moderate, and weak), though no association was detected between ACME genes and strong biofilms or *icaADBC*. In ACME-harboring isolates, *embp* (64%, n = 20), *aap* (67%, n = 21), and *atlE* (61%, n = 19) were significantly prevalent compared to ACME-negative isolates (*P* < 0.010) (Fig. 3).

## Discussion

In therapeutic and infection control strategies, it is important to identify the genetic and phenotypic characteristics of CoNS that cause nosocomial infections, including the presence of virulence genes, biofilm production, and antibiotic resistance [23]. Several characteristics of CoNS are advantageous for the pathogenesis, colonization, and transmission of these bacteria [13, 20]. CoNS are the reservoir of genes in hospitals and are considered in gene dissemination to other bacteria, and healthcare workers have an important role in transmitting these bacteria to hospitalized patients [24, 25]. In addition, MR-CoNS are often resistant to other antibiotics and present challenges in hospitals, especially in intensive care units [3]. In this study, we investigated biofilm formation and identified the biofilm-associated and virulence genes as well as IS*256*, IS*257*, *mecA*, and ACME clusters in CoNS clinical and healthcare workers’ isolates.

Biofilm formation is the most important mechanism of pathogenicity in CoNS isolates [3]. We found a significant difference between clinical and healthcare workers’ isolates in producing strong biofilm (P < 0.001), a finding that agrees with other studies [24, 26]. In addition, the prevalence of *icaADBC* was higher in clinical isolates than in healthcare workers’ isolates (42% vs. 12%) (P < 0.030). our study, similar to some other studies, 90% (n = 34) of strong biofilm-producer isolates were positive for *icaADBC*, which showed an association between ica operon and strong biofilm formation in isolates (P < 0.001) [25, 27, 28].

The co-existence of *icaA* and *icaD* increases *N*-acetyl-glucosaminyl-transferase activity for synthesizing polysaccharide intercellular adhesion (PIA) oligomers and improves biofilm formation [12]. According to earlier studies, PIA is considered a putative candid for vaccine development, though more studies on staphylococcal virulence factors are warranted [16–18]. In this study, 91% (n = 42) of healthcare workers’ isolates were biofilm producers, and 23.8% (10/42) were positive for *icaADBC.* This finding should be considered in preventing and controlling infection in hospitals. Our results showed that 63% (5/8) and 43% (20/46) of wound and blood infection samples were strong biofilm producers associated with *icaAD* and other virulence genes, such as *embp* and *aap*. This finding is not unusual because biofilm formation of Staphylococci on a medical device is common in hospital settings [29, 30]. More than 90% of the clinical isolates of *Staphylococcus epidermidis* with *icaD* and strong biofilm phenotype also carried *mecA* and we observed an association between the presence of the *icaD* and the presence of the *mecA* in these isolates. Other studies have reported the presence of the ica operon genes and the simultaneous presence of the *mecA* gene [24, 25]. Although the presence of *ica*-operon have the key role in strong biofilm producer isolates in our study, other isolates formed biofilm at strong, moderate, or weak levels in non-associated *ica*-operon. Therefore, biofilm production in staphylococci is associated with both *ica* operon and proteins, such as MSCRAMMs, which play an important role in biofilm formation in these bacteria [12, 13].

The adhesive surface proteins have an important role in biofilm formation in associated or non-associated *ica* genes [12]. In our results, *fbe* was detected in 23% of clinical, which was about similar to a study in India (20%) and lower than other studies in Germany (100%), and Iran (89.9%) [5, 31, 32]. Moreover, the frequency of *bhp* (6%) was lower than other studies in Iran (15.3%) and Germany (18.8%), also a study in India indicated none of the strong biofilm formers was positive for *bhp* [5, 31, 32]. In addition, 65% (47 of 72) of strong and moderate biofilm-producer isolates were positive for *aap* in our study. In our findings, *fbe* and *bhp* were detected in strong and moderate biofilm-producer isolates. By contrast, other studies in India and Brazil reported these genes in moderate biofilm-producer isolates or the ubiquitous presence of these genes in strong, moderate, and non-biofilm-producer isolates [5, 13]. In our study, *aap*, a gene encoded accumulating associated protein, was detected in 55% (n = 63) of isolates, this rate is difference from other studies in Iran (94.8% and 64.4%), Germany (93.8%) and India (10%) [5, 31–33]. In the present study, 73% (56 out of 76) of strong and moderate biofilm-producer isolates were positive for *aap*, indicating this protein’s importance in biofilm formation. *aap* was detected in clinical and healthcare workers’ isolates without significant differences. We found that the prevalence of *atlE* (51%) and *embp* (55%) differed from other studies in India and Brazil (30–100%), this discrepancy may be due to different sources of specimens in studies [5, 13]. Among 37 strong biofilm isolates that were positive for *icaA* and *icaD*, 20 (54%) isolates were positive for IS*256*, indicating an association between *icaAD*, biofilm formation, and IS*256*. This finding is similar to the results obtained by Petrelli et al. [34]. In total, the frequency of *icaA*, *icaD*, and IS*256* was significantly higher in MR and clinical isolates in comparison with methicillin-sensitive and healthcare workers’ isolates, which indicates an association among *mecA*, *icaA*, *icaD*, and IS*256*, similar to earlier studies [25, 32, 35]. In addition, our study supported the idea that IS*256* acts as a marker for clinical isolates [4, 25]. In our study, 40% of healthcare workers’ isolates were ACME-positive. The high prevalence of ACME among commensal Staphylococcus species notably affects the growth, survival, colonization, and spread of CoNS among healthcare workers in hospital settings [20]. Also, eight MR-clinical biofilm-producer isolates from blood and wound infections harbored ACME elements, and more than 70% carried *icaADBC, embp, atlE*, and *aap*.

However, CoNS are part of normal skin flora. The presence of virulence factors related to the attachment alone does not confirm the pathogenicity of this bacteria, and other factors should be considered. On the other hand, the presence and expression of a set of genes and environmental factors, such as temperature and osmolality, contribute to pathogenicity and biofilm formation in Staphylococci [36–38]. We detected all studied genetic elements in both clinical and healthcare workers’ isolates. Our results showed that all the ACME-positive isolates were biofilm producers, which increased their survival rate in the hosts. In our study, the high ACME prevalence in healthcare workers’ isolates and its dissemination might contribute to the spread of isolates due to the direct contact of contaminated healthcare workers’ hands with patients.

## Conclusion

In this study, we compared biofilm formation and the frequency of genes involved in biofilm formation, virulence genes, and IS*256*, IS*257*, and ACME in clinical and healthcare workers’ CoNS isolates. We found a significant relationship between clinical isolates and the presence of *icaADBC, mecA*, and IS*256*. We detected some transposable elements contributed to pathogenicity, colonization, and transmission in CoNS isolates. In addition, strains with similar characteristics were found in the clinical and healthcare workers’ isolates, indicating the need for strategies to control and prevent infection in hospitals.

## Materials and methods

### Bacterial strains and methicillin-resistant CoNS detection

In this study, 114 CoNS isolates were collected from clinical samples (n = 57) and volunteer healthcare workers’ skin (n = 57) from January 2019 to December 2021. The healthcare workers included the nurses and physicians at Afzalipour referral hospital in Kerman, Iran. We excluded staff that cleaned their hands with antiseptics at the time of analysis. Clinical samples were isolated from blood, cerebrospinal fluid (CSF), and wounds. Systemic inflammation, absolute neutrophil count (ANC), C-reactive protein (CRP) levels, mono-microbial growth in cultures, and isolate detection from the second blood culture were the laboratory criteria to distinguish true infection and contamination [39]. The isolates were identified using standard biochemical tests, including colony morphology on Baird–Parker agar (Merck, Co, Germany), Gram staining, catalase production, oxidase test, coagulase-negative tests, susceptibility to novobiocin, and the utilization of xylose, arabinose, sucrose, maltose, mannitol, lactose, ribose, fructose, and mannose. Finally, they were confirmed as CoNS by multiplex-PCR, as described by Hirotaki et al. [40]. The multiplex-PCR reaction was performed in a volume of 50 µL containing: 25 µL of Taq DNA Polymerase Master Mix RED (Ampliqon, Co, Denmark), 2 µL DNA template, 0.25 µL of each primer (10pM), and DNase and RNase free water top up to 50 µL. multiplex-PCR amplification was carried out under the following conditions an initial denaturation step 5-min at 95 °C followed by 30 cycles (30 s of denaturation at 95 °C, 30 s of annealing at 58 °C, and 70 s of extension at 72 °C) and a final elongation step at 72 °C for 5 min. *Staphylococcus epidermidis* RP62A was used as a positive control strain in the M-PCR technique.

According to the clinical & laboratory standards institute (CLSI), cefoxitin (FOX, 30 µg) disc was used to screen methicillin-resistant isolates (MR-CoNS) [41]. Then, *mecA* gene was determined using the polymerase chain reaction (PCR) method in MR-CoNS, as previously described by Ruzauskas et al. [42].

### Biofilm assay

Biofilm formation assay was determined according to the microtiter plate method described by Stepanovic et al. [43]. Briefly, The culture of each isolates was prepared in BHI medium overnight and adjusted to 0.5 McFarland. After incubation, the stationary-phase culture is vortexed and diluted 1:100, in medium for bioflim cultivation (TSB supplemented with glucose). The diluted bacteria are vortexed and then inoculated into a microtiter plate (200 mL per well). The microplates were incubated overnight for 24 h at 37 °C. Then, the wells were washed with PBS and fixed with methanol. The wells were stained with crystal violet and then Ethanol 95% were added to each wells. Finally The optical density of each plate was measured at 570 nm. All isolates were classified into the following categories: strong, moderate, weak and non-biofilm producer. The *S. epidermidis* strains ATCC 35,984 (formerly RP62A) was used as positive control.

### Detection of ***ica*** operon, ACME type and virulence genes

Bacterial DNA was extracted according to the boiling method [44]. The presence of *icaA, icaD, icaB, icaC*, *atlE, aap, bhp, embp, and fbe* were determined by the PCR method [13, 25, 32]. The PCR program was run as follows: the initial denaturation at 94 °C for 3 min, 2) 30 cycles of 94 °C for 1 min, 3) annealing temperature (See Table 5) 1 min at 72 °C for extension step, and 4) final extension for 5 min at 72 °C. The products were detected on 1.5% agarose gel, and the band size was compared to a DNA marker (100 bp) Fig. 1. The allotypes of ACME were determined by the PCR [13]. ACME allotypes included ACME-I consisting of the *arc* and the *opp-3* gene clusters, ACME-II containing the *arc*, and ACME-III constituting the *opp-3*. The primers used to identify *ica* operon and virulence genes are presented in Table 5. *Staphlococcus epidermidis* RP62A (ATCC 35,984) was used as positive control for *aap, bhp, embP*, and *ica-*operon genes in the PCR method.

**Table 5:** Target genes, primer sequences, and annealing temperatures used in this study

Target genes	Primer sequence (5´-3´)	PCR product size (bp)	Annealing Tm (˚C) and Time (S)	Reference
***icaA***	F-TCTCTTGCAGGAGCAATCAA R-TCAGGCACTAACATCCAGCA	188	58 ˚C for 30s	(34)
***icaB***	F-ATGGCTTAAAGCACACGACGC R-TATCGGCATCTGGTGTGACAG	526	50 ˚C for 30s	(24)
***icaC***	F-ATCATCGTGACACACTTACTAACG R-CTCTCTTAACATCATTCCGACGCC	934	50 ˚C for 30s	(24)
***icaD***	F-ATGGTCAAGCCCAGACAGAG R-CGTGTTTTCAACATTTAATGCAA	198	55 ˚C for 30s	(34)
***aap***	F-ATACAACTGGTGCAGATGGTTG R-GTAGCCGTCCAAGTTTTACCAG	400	50 ˚C for 30s	(13)
***atlE***	F-CAACTGCTCAACCGAGAACA R-TTTGTAGATGTTGTGCCCCA	682	62 ˚C for 60s	(13)
***bhp***	F-ACGGACAATATCGTCTCTCAA R-AACTTCAGCCGTTCCCTT	1917	55 ˚C for 30s	(13)
***embp***	F-AGCGGTACAAATGTCAATATC R-AGAAGTGCTCTAGCATCATCC	455	62 ˚C for 60s	(13)
***fbe***	F-TAAACACCGACGATAATAACCAAA R-GGTCTAGCCTTATTTTCATATTCA	496	62 ˚C for 60s	(13)
***arcA***	F-CTAACACTGAACCCCAATG R-GAGCCAGAAGTACGCGAG	1946	52 °C, 1 min;	(13)
***opp3AB***	F-GCAAATCTGTAAATGGTCTGTTC R-GAAGATTGGCAGCACAAAGTG	1183	52 °C, 1 min;	(13)

### IS***256*** and IS***257*** detection

The IS*256* (1103-bp) and IS*257* (621-bp) were detected by the PCR technique described by Kozitskaya et al. [4] The F-5′-TGAAAAGCGAAGAGATTCAAAGC and R-5′-ATGTAGGTCCATAAGAACGGC primers were used for IS*256*, and the primer sequences of F-5′-GCTAATTTCGTGGCATGGCG and R-5′- GTTATCACTGTAGCCGTTGG were used for IS*257.* The PCR program was run based on the following steps: (1) initial denaturation at 94 ºC for 4 min, (2) 30 cycles of denaturation at 92 ºC for 30s, (3) annealing step at 60 ºC for 40s, (4) elongation at 72 ºC for 30s, and (5) final extension at 72 ºC for 5 min. The product was visualized on 1.5% agarose gel, and the band size was compared to a DNA marker (100 bp).

### Statistical analysis

We analyzed the data on SPSS version 23 (IBM, Armonk, NY, USA). The chi-square and Fisher’s exact tests were used to compare categorical variables, and *P* values ≤ 0.05 were considered statistically significant.

### Electronic supplementary material

Below is the link to the electronic supplementary material.

Supplementary Material 1

## Data Availability

The datasets used and/or analyzed during the study are available on reasonable requests from the corresponding author.

## Abbreviations

CoNS: Coagulase negative staphylococci
MR-CoNS: Methicillin-resistant CoNS
ACME: Arginine catabolic mobile element
*fbe*: Fibrinogen binding protein
*bhp*: Bap homologous- protein
*embp*: Extracellular matrix-binding protein
*aap*: Accumulation-associated protein
MSCRAMMs: Microbial surface components recognizing adhesive matrix molecules
PIA: Polysaccharide intercellular adhesion
PNAG: Poly-N-acetyl-glucosamine
NICU: Neonatal intensive care units
ICU: Intensive care unit
ID: Infectious disease units
PICU: Pediatric intensive care units
BMT: Bone marrow transplant
