# Supplementary material for: The frequency of adherence, biofilm-associated, Arginine Catabolic Mobile element genes, and biofilm formation in clinical and healthcare worker coagulase-negative staphylococci isolates
Source: BMC Microbiol. 2023 Aug 15;23:222. doi: 10.1186/s12866-023-02959-x (PMC10426181; doi:10.1186/s12866-023-02959-x)
Supplement: Supplementary file 1 — Supplementary Material 1 [file 12866_2023_2959_MOESM1_ESM.pdf]

Supplementary information with changes marked (PCR electrophoresis images)

PCR electrophoresis images for virulence, ACME genes, and IS (Figures 1-1, 2-1, 1-2, and 2-2).

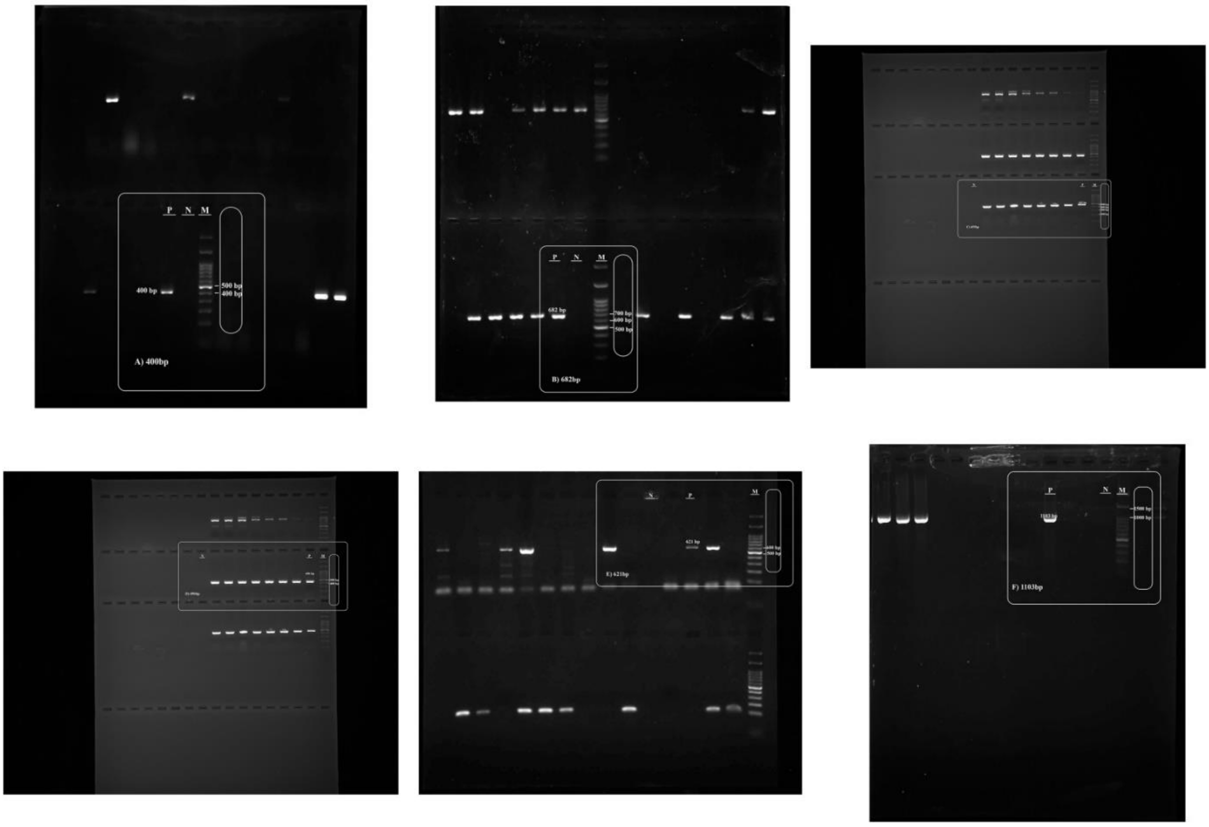

Figure 1-1. PCR electrophoresis: A: *aap* (400 bp), B: *atlE* (682 bp), C: *embp* (455 bp), D: *fbe* (496 bp), E: IS*257* (621 bp) and F: IS256 (1103 bp) F: M: 100 bp DNA marker, P: Positive control, N: Negative control. The background light has decreased, contrast is increased and Light adjusted.

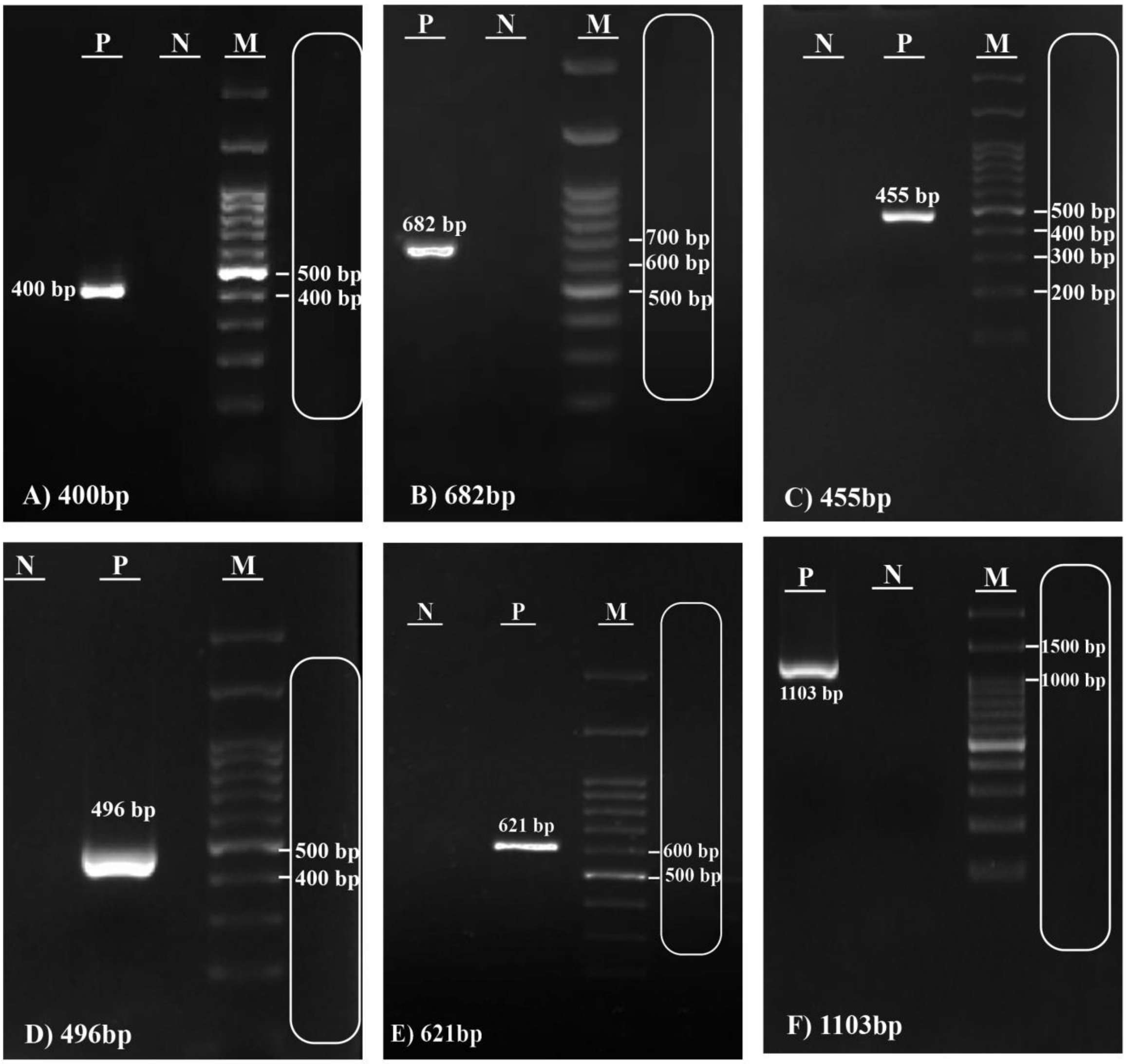

Figure 2-1. PCR electrophoresis: A: *aap* (400 bp), B: *atlE* (682 bp), C: *embp* (455 bp), D: *fbe* (496 bp), E: IS*257* (621 bp) and F: IS256 (1103 bp) F: M: 100 bp DNA marker, P: Positive control, N: Negative control. The background light has decreased, contrast is increased and Light adjusted.

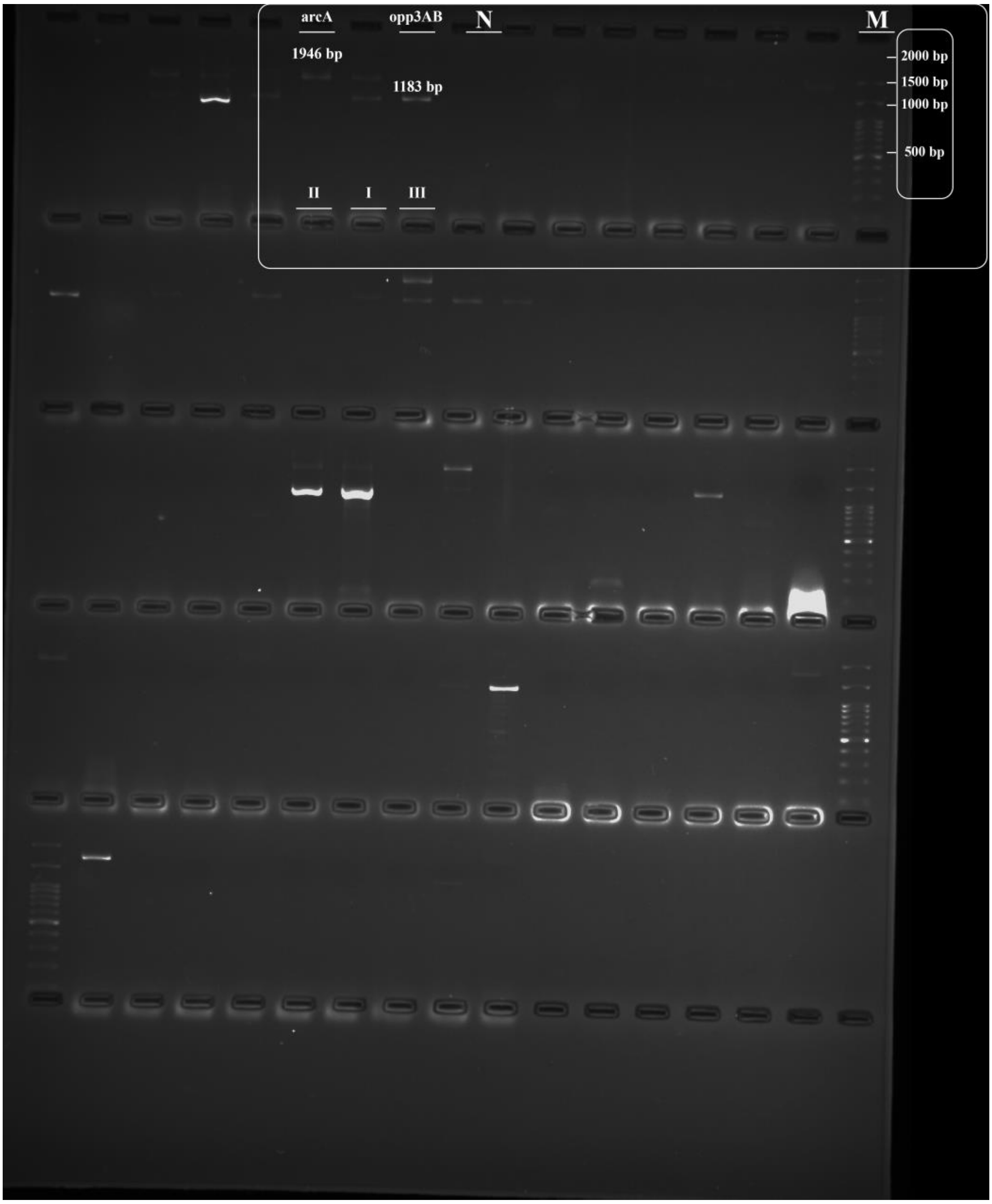

Figure 1-2. PCR electrophoresis: *opp3AB* (1183 bp) and *arcA* (1946 bp), M: 100 bp DNA marker. I: ACME- I, II: ACME- II, and III: ACME- III. N: Negative control. The background light has decreased, contrast is increased and Light adjusted.

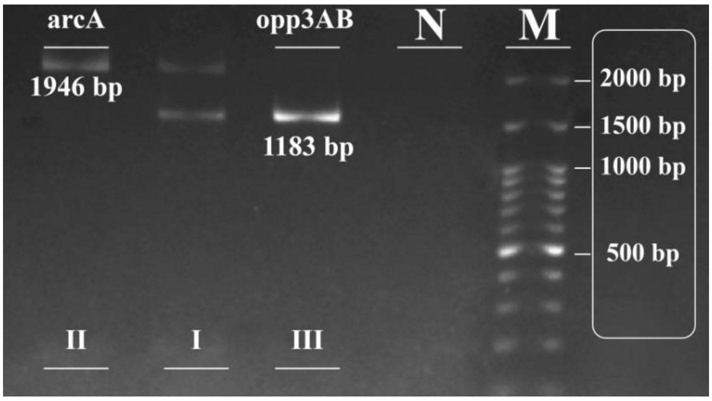

Figure 2-2. PCR electrophoresis: *opp3AB* (1183 bp) and *arcA* (1946 bp), M: 100 bp DNA marker. I: ACME- I, II: ACME- II, and III: ACME- III. N: Negative control.
